# Comparison of Virtual Reality-Assisted Visual Training with Conventional Strategies in the Treatment of Bilateral Refractive Amblyopia

**DOI:** 10.3390/children12040447

**Published:** 2025-03-31

**Authors:** Hsiu-Mei Huang, Yu-Ting Hsiao, Yi-Hao Chen, I-Hui Yang

**Affiliations:** 1 Department of Ophthalmology, Kaohsiung Chang Gung Memorial Hospital and Chang Gung University College of Medicine, Kaohsiung 833401, Taiwan; sammy1013@cgmh.org.tw (H.-M.H.); yuting1008@cgmh.org.tw (Y.-T.H.); ophkh@cgmh.org.tw (Y.-H.C.); 2 Department of Ophthalmology, Kaohsiung Municipal Fong Shan Hospital-Under The Management of Chang Gung Medical Foundation, Kaohsiung 830025, Taiwan

**Keywords:** refractive amblyopia, visual training, Cambridge Visual Stimulator, virtual reality assisted training

## Abstract

Background: Visual training based on perceptual learning and dichoptic stimulation has been developed for amblyopic treatment. However, reports on virtual reality-assisted (VR) training for amblyopia are limited. The aim of this study was to evaluate the effects of VR training and compare the visual outcomes of different treatments in children with bilateral refractive amblyopia. Methods: Children aged 4–8 years with a best-corrected visual acuity (BCVA) less than 20/25 were included. All of the patients had worn glasses for at least 2 months before enrollment. At enrollment, age, gender, refractive status, baseline BCVA, and amblyopia severity were recorded. The treatment outcomes were evaluated in terms of BCVA at 24 weeks, the change in BCVA from baseline to 24 weeks, and the time taken to achieve treatment success (BCVA ≥ 20/25). Results: The longitudinal study included 30 patients in the Cambridge Visual Stimulator (CAM) training group, 14 in the VR training group, and 26 in the control group (glasses only). Among patients with an initial BCVA between 20/100 and 20/40, a significantly higher proportion of those in the VR training group achieved treatment success compared to the control group (*p* = 0.022). Among those who achieved treatment success, the time taken to achieve success was significantly shorter in the CAM and VR training groups compared to the control group (*p* < 0.001). Conclusions: Visual training can shorten the time taken to achieve treatment success compared to glasses alone. While VR training seems more effective than CAM training, both are valuable strategies for amblyopic children who respond poorly to glasses alone.

## 1. Introduction

Amblyopia is the most common cause of visual impairment in children and has a lifelong and profound impact [1]. Typically, amblyopia is defined as reduced best-corrected visual acuity (BCVA) in one or both eyes. The mechanism of amblyopia is recognized as inadequate visual experience in the visual pathway without anatomical abnormalities during early life. Therefore, early screening and treatment in children are necessary to prevent permanent visual loss in amblyopic eyes. The causes of amblyopia are categorized into strabismus, anisometropia, high bilateral refractive errors, and visual deprivation. The treatments for amblyopia are aimed at (1) eliminating the underlying causes, such as correcting ocular misalignment in strabismus, removing the form deprivation sources, and correcting refractive errors; and (2) penalizing the unaffected eye by occlusion therapy, pharmacological or optical penalization methods [2]. The Pediatric Eye Disease Investigator Group (PEDIG) found that nearly one-third of amblyopic children did not have amblyopia after treatment with optical correction alone [3]. However, approximately 15–50% of children with amblyopia, whether unilateral [4,5] or bilateral [6,7], do not achieve normal visual acuity (VA) despite extended periods of treatment, indicating that conventional treatment is inadequate. In recent years, amblyopia has been recognized as a binocular disorder involving interocular suppression, leading to increased research on binocular dysfunctions. Based on this, new therapies, such as perceptual learning and dichoptic visual training, have been developed to balance visual input from both eyes, reduce interocular suppression, and enhance simultaneous perception.

The concept of perceptual learning has been applied to advanced amblyopia therapy by encouraging amblyopic eyes to perform fine visual discriminations with repeated practice [5]. Cambridge Visual Stimulator (CAM), the first application of perceptual learning, was reported to be effective for improving visual performance in children and adults with amblyopia [8,9].

Dichoptic visual training, which stimulates both eyes simultaneously and reduces the suppression of the amblyopic eye [10], has recently been shown to improve visual function in amblyopic eyes [10,11]. With the evolution of digital devices, virtual reality (VR)-assisted training based on dichoptic stimulation has been applied and revealed to be effective for improving VA in amblyopic adults [12]. Although some positive effects were reported in this training, there was no report comparing the effects of different treatments for amblyopia. Bilateral amblyopia results from cortical suppression due to blurred retinal input, but related studies are limited, with small sample sizes and short follow-up periods. Therefore, this study aimed to investigate the visual outcomes after different treatments in bilateral amblyopic children.

## 2. Materials and Methods

### 2.1. Patient Enrollment

This longitudinal and case-control study was performed in Kaohsiung Chang-Gung Memorial Hospital, a tertiary medical center in southern Taiwan. Based on chart review, patients aged at least 4 years and younger than 8 years with a diagnosis of ametropic amblyopia between January 2015 and June 2023 were enrolled. Ametropic amblyopia is defined as an initial BCVA < 20/25 as determined using the Landolt C chart associated with the presence of or a history of high refractive errors in both eyes and no interocular difference in the BCVA of two lines. Based on the level of refractive errors, high hyperopia was defined as ≥+3.5 diopters (D), high myopia was myopia ≤ −5.0 D, and high astigmatism was ≤−2.0 D. The severity of amblyopia was classified according to the initial BCVA: worse than 20/100 was severe, better than 20/40 was mild, and other scores were moderate amblyopia [3]. The exclusion criteria included children with anisometropia, strabismus, learning difficulties, developmental delays, and ocular or coexisting systemic diseases contributing to their impaired vision. All of the enrollees completed a minimum follow-up of at least 6 months. The study was approved by the institutional review board in the Kaohsiung Chang-Gung Memorial Hospital (IRB number: 202101080B0) and adhered to the Declaration of Helsinki.

### 2.2. Treatment for Amblyopia

**Figure 1:**
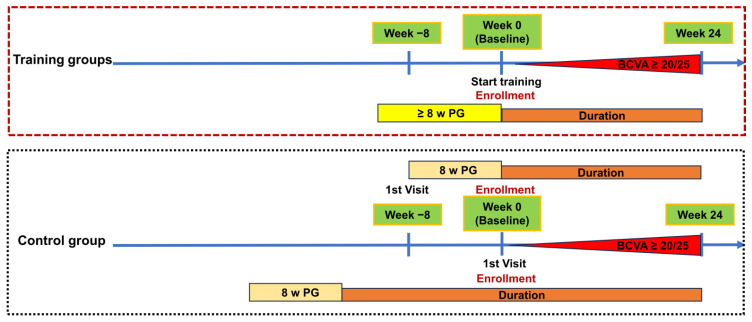
Schematic illustration of the study design. All the patients had worn glasses for at least 8 weeks before enrollment. The time of enrollment (Week 0) was defined as the time of starting training in the training groups. For the control group, it was either the time of their first visit to our hospital or 2 months thereafter (depending on the duration of PG wearing at the time of their first visit to our hospital). The time taken (duration) to achieve BCVA ≥ 20/25 (treatment success) and to reach 24 weeks (Week 24) was calculated from the time of enrollment (Week 0) in the training groups, while it was calculated from the time after wearing glasses for 8 weeks in the control group. PG: prescribed glasses wearing; w: weeks.

At the first visit, data from complete ophthalmological examinations, including BCVA, cycloplegic refractions, slit-lamp examinations, fundoscopy, ocular movements, and alignment, were recorded. Because cyclopentolate was not available in our hospital, and the cycloplegic effect of tropicamide was non-inferior to cyclopentolate [13], cycloplegic refractions were performed 20 min after instilling 1% tropicamide eye drops (Alcon, TX, USA), one drop every 10 min, for a total of three times. The prescriptions for worn glasses were fully corrected for cycloplegic refractive errors with a spherical equivalent (SE) ≥ 1.00 D or ≤−1.00 D. Patients who could not or refused to attend training were assigned to the control group to wear glasses alone full-time. Patients enrolled before November 2021 received CAM training, while those enrolled after December 2021 received VR training. In the training groups, the enrolled patients had worn glasses full-time for at least 8 weeks before starting these trainings and continued to wear glasses during and after the training sessions. The timeline for the treatment protocols of these groups is shown in Figure 1. Treatment success was defined as a BCVA ≥ 20/25 before or at Week 24. The baseline for the analysis was established as the point when patients in the training groups started their training (Week 0) or after patients in the control group had worn glasses for a duration exceeding 8 weeks. The time taken to achieve treatment success and the final BCVA at 24 weeks were recorded.

#### 2.2.1. Training Programs Based on Perceptual Learning

The CAM training program for our CAM group was binocular and included both CAM and cheiroscope trainings. CAM is a device for amblyopic training based on the theory of perceptual learning. During CAM training, the patient was shown a series of high-contrast square-wave gratings of different spatial frequencies at a distance of approximately 28 cm from the apparatus, and training was undertaken with the three smallest discernible gratings. Each, in turn, was rotated at one revolution per minute behind a clear Perspex cover on which the patient drew or played pencil games under supervision [14]. A cheiroscope is an optical device comprising a viewing instrument paired with a drawing pad. The viewing instrument, configured as a haploscope, which seamlessly blends an image perceived by one eye into the drawing perspective of the other eye, is a tool for training binocular vision and enhancing eye-hand coordination [15]. The selection of images depended on the severity of amblyopia. The more severe the amblyopia, the simpler the images were that were chosen for training in the beginning. Each session of training lasted 15–30 min, was followed by recording BCVA, and was repeated weekly.

#### 2.2.2. Dichoptic Visual Training Assisted with Virtual Reality (VR) Device

Prior to training, the enrolled patients and their parents were informed of the possible side effects of VR-assisted training with a head-mounted device (HMD), such as short-term fatigue or visual discomfort after the use of the immersive device. The VR-assisted dichoptic visual training (VR training) was performed using the 2.73 version of the computer game Diplopia Game (Vivid Vision, CA, USA), which was run on an HTC virtual reality head-mounted display (HTC Vive, TWN). The HTC Vive was equipped with an AMOLED display (5,7″diagonal; resolution of 960 × 1080 pixels per eye; 100° field of view), mounted with a sensor for the positional tracking system, and connected to a personal computer system. Seven games were available, and the games for anti-suppression with a dichoptic setting were selected to train the brain to use both eyes simultaneously to play. Our VR training program comprised a weekly training session lasting 30–40 min that included two different games (15–20 min per game). One game trained one eye, while the other game trained the other eye. The training was stopped if BCVA reached ≥ 20/25 or if the training had been undertaken for 24 weeks.

### 2.3. Statistical Analysis

Statistical analyses were performed using a commercially available statistical software (SPSS for Windows, version 20.0, IBM Corp, Armonk, NY, USA). Descriptive statistics were used to characterize the training and control groups. For statistical analysis, the BCVA as determined using the Landolt C chart was converted to the logarithm of the minimum angle of resolution (logMAR). The improvement in BCVA from baseline to 24 weeks was analyzed using paired-samples t-tests. The chi-square test was used to compare the groups in terms of categorical variables such as gender, amblyopia severity, the status of refractive errors, and the proportion of participants in each group who achieved treatment success. Independent t-tests were used to compare continuous variables between the treatment success and non-success groups. One-way ANOVA followed by post-hoc Bonferroni comparison tests or repeated-measures ANOVA was used to analyze the differences in continuous variables between groups. The factors associated with treatment success were analyzed by using logistic regression. A *p*-value less than 0.05 was considered statistically significant for all the tests.

## 3. Results

### 3.1. Patient Characteristics

The clinical characteristics of the study population were summarized in Table 1. A total of 70 patients were enrolled and assigned to the CAM (n = 30), VR (n = 14), or control groups (n = 26). No discomfort or serious adverse events potentially related to visual training, such as headaches, dizziness, eye strain or pain, blurred vision, or dry eyes [16], were reported in any of the patients during training, after training sessions, or during the follow-up period.

The mean age of the patients at enrollment was 4.82 ± 0.89 years. The mean BCVA of the amblyopic eye at baseline was 0.32 ± 0.14 logMAR, 0.27 ± 0.09 logMAR, and 0.37 ± 0.21 logMAR in the CAM, VR, and control group, respectively. Regarding the distribution of refractive errors, the majority of the patients had high astigmatism (≤−2.0 D) in all three groups: 53.3%, 42.9%, and 57.7% in the CAM, VR, and control groups, respectively. Moreover, there were more patients with high astigmatism combined with high myopia (≤−5.0 D) in the VR group compared to its counterparts. The demographic variables with regard to age, gender, baseline BCVA and the severity of amblyopia did not differ between the three groups. However, the distributions of the refractive error associated with amblyopia were significantly different among the three groups (*p* = 0.027). Subgroup analysis using the chi-square test revealed a significant difference in the distribution of the refractive error between the CAM group and the control group (*p* = 0.036), but not between the VR group and CAM or control groups.

### 3.2. Treatment Outcomes

The mean BCVA of the amblyopic eye at 24 weeks was 0.11 ± 0.14 logMAR in the CAM group, 0.05 ± 0.05 logMAR in the VR group, and 0.13 ± 0.16 logMAR in the control group. Among all the enrolled patients, their final BCVA at 24 weeks was significantly better than their baseline BCVA (*p* < 0.001). Treatment success was achieved in 21 (70%), 13 (92.9%), and 14 (53.8%) patients of the CAM, VR, and control groups, respectively. The mean change in the logMAR BCVA from baseline to 24 weeks did not differ significantly between the three groups (*p* = 0.155). A significantly higher proportion of patients in the VR group had treatment success than in the control group (*p* = 0.012) (Table 1).

### 3.3. Analysis for Moderate Amblyopia

Moderate amblyopia (BCVA 0.3–0.7 logMAR) was diagnosed in 18 (60%), 7 (50%), and 15 (57.7%) patients in the CAM, VR, and control groups, respectively. Treatment success was achieved in 12 (66.7%), 6 (85.7%), and 5 (33.3%) patients of the CAM, VR, and control groups, respectively. There was no significant difference in the mean change in logMAR BCVA from baseline to 24 weeks between the three groups (*p* = 0.179). A significantly higher proportion of patients in the VR group achieved treatment success than in the control group (*p* = 0.022) (Table 2).

### 3.4. Factors Influencing Treatment Success

The BCVA of the success group at 24 weeks (48 patients, 68.6%, 0.03 ± 0.05 logMAR) was significantly better than that of the non-success group (22 patients, 31.4%, 0.26 ± 0.15 logMAR) (*p* < 0.001) (Table 3). The mean logMAR BCVA of the amblyopic eye at baseline in the success group (0.27 ± 0.09 logMAR) was significantly lower than that in the non-success group (0.46 ± 0.20 logMAR) (*p* < 0.001), and a higher proportion of patients receiving VR training achieved treatment success, indicating that treatment success was strongly correlated with BCVA at baseline (*p* = 0.001) and treatment groups (*p* = 0.027, Table 1). In the treatment success group, the mean time taken to achieve success was 14.19 ± 6.42 weeks for the CAM group (*p* < 0.001) and 6.92 ± 5.22 weeks for the VR group (*p* < 0.001), both of which were significantly shorter than that of the control group (54.74 ± 47.12 weeks) (Table 4). The sub-analysis of patients with moderate amblyopia showed that treatment success was also significantly correlated with BCVA at baseline (*p* = 0.009) and treatment groups (*p* = 0.039, Table 2). The mean time taken to achieve success was 15.17 ± 5.64 weeks for the CAM group (median, 15 weeks; range, 5–24 weeks; *p* < 0.001) and 11.5 ± 4.14 weeks for the VR group (median, 12.5 weeks; range, 6–16 weeks; *p* = 0.001), both of which were significantly shorter than that of the control group (56.11 ± 34.48 weeks; median, 36 weeks; range, 24–95 weeks) (Table 4).

## 4. Discussion

This is a pilot study to compare the effects of different treatment strategies for bilateral refractive amblyopia. Our study shows that initial BCVA and whether training was conducted determine the success of amblyopia treatment. Training, either CAM or VR, is effective in accelerating improvement and increasing the success of treatment for bilateral refractive amblyopia.

According to the randomized trials conducted by the PEDIG, children with moderate amblyopia received spectacle correction for at least 4 weeks before enrollment [17,18]. Meanwhile, their later study found that more than 80% of the patients achieved their best measured acuity after 8–12 weeks of wearing spectacles only [19]. Therefore, to minimize the influence of optical correction alone on amblyopia treatments, the enrollees in our study were required to wear fully corrected spectacles for a minimum of 8 weeks.

Individuals with bilateral refractive amblyopia compose a relatively small subgroup of amblyopic children, and the VA is generally considered to be well improved after optical correction treatment. Earlier and smaller retrospective studies reported that children with bilateral hyperopic amblyopia achieved a final binocular VA of 20/25 in approximately 48% of cases after a mean follow-up of 40–55 months [7,20]. Wallace et al. reported that a BCVA ≥ 20/25 was achieved at 26 weeks in 59% of 113 children (aged 3–10 years; mean, 5.1 years) with moderate (VA ≤ 20/40) and hyperopic amblyopia (SE ≥ +4.0 D) [21]. Chen et al. reported a final VA ≥ 20/25 at a mean of 28.8 months in 74.7% of 217 Asian children (3–10 years old, mean 6 years) with hyperopic (SE ≥ +4.0 D) amblyopia and found that their VA greatly improved within 12 months [22]. In our study, only 33.3% of moderate amblyopic patients (mean age 4.47 years) in the control group achieved a BCVA ≥ 20/25 at 24 weeks, which is lower than that in previous reports. The speculated reason is that the majority of the patients in our control group (66.6%) had high astigmatism (≤−2.0 D), whereas most of the amblyopic patients in previous studies were highly hyperopic. This indicates that patients with bilateral amblyopia caused by high astigmatism have relatively poor responses to optical correction alone and require other strategies for achieving treatment success.

The reason for the inadequate response to conventional amblyopia treatment remains unclear, but it is presumed to be the decorrelation of binocular visual experience and habitual suppression of the amblyopic eyes. Therefore, strategies to improve vision in amblyopia by stimulating binocular function have been developed [5]. Visual training consists of orthoptic training (comprising dichoptic training) and perceptual learning training to improve binocular visual function [6].

Perceptual learning, defined as a consistent change in the perception of a stimulus array following practice [23], strengthens the visual function of amblyopic eyes after intensive and repeating similar activities [5]. Several studies have shown that visual training with perceptual learning improved visual performance in tasks such as contrast sensitivity and letter recognition in children and adults with anisometropic amblyopia by inducing plasticity in the visual system [8,9]. Meanwhile, monocular training with perceptual learning improved not only the monocular function of amblyopic eyes, but also the binocular combinations [24]. The first clinical application of perceptual learning theory, CAM, was conducted by passively viewing high-contrast rotating sine wave gratings with monocular amblyopic eyes [25]. Our previous study reported that CAM and a cheiroscope are effective for helping 3- to 7-year-old children with bilateral refractive amblyopia to reach VA ≥ 20/25 within 12 weeks [26]. Huang et al. also reported that a visual training program including perceptual learning was successful in improving VA and stereoacuity in 7- to 10-year-old children with bilateral amblyopia who responded poorly to conventional treatment [6]. In this study, more patients with moderate amblyopia in the CAM group (66.7%) achieved treatment success at 24 weeks as compared to the control group (33.3%). The time interval for treatment success was significantly shorter in the CAM group (median, 15 weeks) than in the control group (median, 36 weeks; *p* < 0.001). These data suggest that CAM training programs are an effective strategy for shortening the duration of amblyopia treatment in children.

Given the belief that amblyopia involves abnormal binocular interactions and interocular suppression, recent studies have reported the benefit of dichoptic training in the treatment of amblyopia. Dichoptic training involves stimulating both eyes simultaneously, with lower-contrast stimuli presented to the fellow eye to counteract its suppression of the amblyopic eye. This approach enhances neural plasticity more effectively than monocular training of the amblyopic eye alone, leading to better therapeutic outcomes [10]. Dichoptic training through playing binocular games improved VA in amblyopic children and adults [10,11,27,28]. With the advancement of technology, VR has been applied to be a new, safe, and effective method in the field of neurorehabilitation [29,30,31]. VR-assisted amblyopic training makes use of different games designed based on perceptual learning and dichoptic stimulation to promote improvements in VA, contrast sensitivity, and stereopsis in amblyopic eyes [32]. A small pilot study demonstrated significant improvements in VA and stereopsis in adults with amblyopia after eight 40-min training sessions [12]. In our study, more than 85% of the patients aged 4–9 years in the VR training group acquired BCVA ≥ 20/25 within 13 weeks of training. Compared to the control group, a significantly higher proportion of patients in the VR group achieved treatment success. Meanwhile, the time taken to achieve treatment success was prominently shorter in the VR group than in the control group. These results indicate that visual training assisted by VR is effective for promoting and accelerating VA improvement in children with bilateral amblyopia.

The response to amblyopia treatment depends on many factors, including the initial VA, the causes and severity of amblyopia, the duration of the abnormal visual experience, the method of amblyopia therapy, the age at which treatment is started, the duration of treatment, the level of compliance, and the speed at which the treatment is tapered [33]. We found that the initial BCVA, the severity of amblyopia, and whether training was conducted were the important determinants of treatment success at 24 weeks. In this study, VR training seemed to be more efficient than CAM training, presumably because the interactive VR programs are more appealing to children, thus resulting in better compliance and outcomes.

Our study has several limitations. These included its retrospective nature, with possible recall bias, and the scarcity of bilateral amblyopic patients, leading to a small sample size. Moreover, some studies indicate that approximately 60% of patients with bilateral refractive amblyopia acquired a BCVA > 20/25 at 26 weeks [21] and that most of the visual improvement occurred within 36 weeks after optical correction [34]. Our follow-up time of 24 weeks may be valid, but it is limited for explaining the final visual outcome. Since VR-assisted training was only made available after December 2021, and assignment to the training group was based on individual preference, selection bias could exist, and thus the results from this study should be interpreted carefully. Nonetheless, a comparison of baseline demographic data (Table 1) indicates that the control and training groups were similar in terms of age, gender, and baseline BCVA, supporting the validity of the three-group comparison results. Further studies with randomized prospective designs, larger populations, and longer follow-up times are needed to verify the results of this study. The high cost and limited availability of VR equipment, coupled with the requirement for patient supervision during training, meant that training could only be conducted in medical institutions. These challenges hinder the wide implementation of VR training programs. The development of home-based devices integrated with remote monitoring could improve compliance with VR training, leading to better outcomes for amblyopia treatment.

## 5. Conclusions

This study is a pilot study and the first to demonstrate the benefits of VR-assisted visual training and to compare the effects of different strategies for treating bilateral refractive amblyopia in children. VR using a binocular visual training device in weekly 30-min sessions is effective for shortening the time taken to achieve treatment success within a median of 13 weeks. Considering the stress and burden associated with poor responses to optic correction alone in children with bilateral refractive amblyopia, our study has important implications for offering these patients potentially better treatment options.

## Figures and Tables

**Table 1:** Clinical Characteristics of enrolled patients with bilateral refractive amblyopia.

Overall	CAM Training	VR Training	Control	*p* Value
Number of patients	30	14	26	
Age (years, mean ± SD)	5.02 ± 0.91	4.79 ± 0.89	4.62 ± 0.85	0.245
Gender (no of male, %)	14 (46.7%)	6 (42.9%)	15 (57.7%)	0.6
Baseline BCVA (logMAR, mean ± SD)	0.32 ± 0.14	0.27 ± 0.09	0.37 ± 0.21	0.206
Severe (<20/100)	0	0	1 (3.8%)	
Moderate (≥20/100 and ≤20/40)	18 (60%)	7 (50%)	15 (57.7%)	
Mild (>20/40)	12 (40%)	7 (50%)	10 (38.5%)	
Refractive errors (n)				0.027 #
Hypermetropia ≥ +3.5 D	1 (3.3%) 1 + 0	2 (14.3%) 2 + 0	7 (26.9%) 4 + 3	
Myopia ≤ −5.0 D	1 (3.3%) 1 + 0	1 (7.1%) 1 + 0	1 (3.8%) 1 + 0	
Astigmatism ≤ −2.0 D	16 (53.3%) 6 + 10	6 (42.9%) 2 + 4	15 (57.7%) 8 + 7	
Hypermetropia + Astigmatism	9 (30%) 7 + 2	1 (7.1%) 0 + 1	3 (11.5%) 2 + 1	
Myopia + Astigmatism	3 (10%) 3 + 0	4 (28.6%) 2 + 2	0 (0%) 0 + 0	
BCVA at 24 weeks (logMAR, mean ± SD)	0.11 ± 0.14	0.05 ± 0.05	0.13 ± 0.16	0.204
Treatment Success	21 (70%)	13 (92.9%)	14 (53.8%)	0.039 *

Treatment Success: BCVA ≥ 20/25 at 24 weeks. Subgroup analysis #: CAM training vs. Control (*p* = 0.036); *: VR training vs. Control (*p* = 0.012).

**Table 2:** Clinical Characteristics of patients with bilateral moderate amblyopia.

Moderate Amblyopia	CAM Training	VR Training	Control	*p* Value
Number of patients	18	7	15	
Age (years, mean ± SD)	4.92 ± 0.97	4.71 ± 1.25	4.47 ± 0.73	0.407
Gender (no of male, %)	8 (44.4%)	5 (71.4%)	9 (60%)	0.422
Baseline BCVA (logMAR, mean ± SD)	0.40 ± 0.13	0.36 ± 0.05	0.45 ± 0.13	0.195
Refractive errors (n)				0.144
Hypermetropia ≥ +3.5 D	1 (5.6%)	2 (28.6%)	4 (26.7%)	
Myopia ≤ −5.0 D	1 (5.6%)	1 (14.3%)	1 (6.7%)	
Astigmatism ≤ −2.0 D	6 (33.3%)	2 (28.6%)	8 (53.3%)	
Hypermetropia + Astigmatism	7 (38.9%)	0 (0%)	2 (13.3%)	
Myopia + Astigmatism	3 (16.7%)	2 (28.6%)	0 (0%)	
BCVA at 24 weeks (logMAR, mean ± SD)	0.13 ± 0.17	0.06 ± 0.06	0.16 ± 0.12	0.313
Treatment Success	12 (66.7%)	6 (85.7%)	5 (33.3%)	0.039 *

Treatment Success: BCVA ≥ 20/25 at 24 weeks. Subgroup analysis *: VR training vs. Control (*p* = 0.022).

**Table 3:** Factors influencing treatment success in patients with bilateral refractive amblyopia.

	Success	Nonsuccess	*p* Value
Number of patients	48 (68.6%)	22 (31.4%)	
Age (years, mean ± SD)	4.76 ± 0.87	4.95 ± 0.95	0.41
Gender (no of male, %)	26 (51%)	9 (47.4%)	0.788
Baseline BCVA (logMAR, mean ± SD)	0.27 ± 0.09	0.46 ± 0.20	<0.001
Severe (<20/100)	0 (0%)	1 (4.5%)	
Moderate (≥20/100 and ≤20/40)	23 (47.9%)	17 (77.3%)	
Mild (>20/40)	25 (52.1%)	4 (18.2%)	
Refractive errors (n)			0.575
Hypermetropia ≥ +3.5 D	5 (10.4%)	5 (22.7%)	
Myopia ≤ −5.0 D	2 (4.2%)	1 (4.5%)	
Astigmatism ≤ −2.0 D	28 (58.3%)	9 (40.9%)	
Hypermetropia + Astigmatism	9 (18.8%)	4 (18.2%)	
Myopia + Astigmatism	4 (8.3%)	3 (13.6%)	
BCVA at 24 weeks (logMAR, mean ± SD)	0.03 ± 0.05	0.26 ± 0.15	<0.001
Treatment Groups (n)			0.039
CAM training	21 (70%)	9 (30%)	
VR training	13 (92.9%)	1 (7.1%)	
Control	14 (53.8%)	12 (46.2%)	

**Table 4:** The duration to reach treatment success in patients with bilateral refractive amblyopia.

	Overall			Moderate Amblyopia		
	n	Weeks to Reach Success (mean ± SD, Median, Range)	*p* Value (vs. Control)	n	Weeks to Reach Success (mean ± SD, Median, Range)	*p* Value (vs. Control)
CAM training	21	14.19 ± 6.42 (14, 5–24)	<0.001	12	15.17 ± 5.64 (15, 5–24)	<0.001
VR training	13	6.92 ± 5.22 (5, 2–16)	<0.001	6	11.5 ± 4.14 (12.5, 6–16)	0.001
Control	14	54.74 ± 47.12 (32, 1–159)		5	56.11 ± 34.48 (36, 24–95)	

## Data Availability

Data are available on reasonable request.
